# Impact and safety of remote monitoring of heart failure patients managed with the HeartLogic algorithm: the HeartLogic France Cohort Study

**DOI:** 10.1093/ehjdh/ztaf133

**Published:** 2025-11-13

**Authors:** Rodrigue Garcia, Daniel Gras, Jacques Mansourati, Pascal Defaye, Arnaud Bisson, Serge Boveda, Lisa Durocher, Estelle Gandjbakhch, Matthieu Gras, Jean-Pierre Gueffet, Caroline Himbert, Peggy Jacon, Pierre Khattar, Benoit Lequeux, Anthony Li, Vincent Mansourati, Damien Minois, Eloi Marijon, Bertrand Pierre, Stéphanie Ragot, Vincent Probst, Bruno Degand

**Affiliations:** Cardiology Department, University Hospital of Poitiers, 2 rue de la Milétrie, Poitiers F-86021, France; Centre D'Investigation Clinique 1402, University Hospital of Poitiers, 2 rue de la Milétrie, Poitiers F-86021, France; Cardiology Department, Hôpital Privé du Confluent, 2-4 Rue Éric Tabarly, Nantes 44000, France; Cardiology Department, University Hospital of Brest, Boulevard Tanguy Prigent, Brest 29200, France; Cardiology Department, University Hospital Grenoble Alpes, Avenue des Maquis du Grésivaudan, Grenoble 38043, France; Cardiology Department, University Hospital of Tours, Avenue de la République, Chambray-lès-Tours 37170, France; Cardiology Department, University Hospital of Orléans, 14 Avenue de l'Hôpital, Orléans 45100, France; Cardiology Department, Clinique Pasteur, 45 Avenue de Lombez, Toulouse 31076, France; Universiteit Ziekenhuis, Vrije Universiteit Brussel (VUB), Laarbeeklaan 101, Jette Brussels 1090, Belgium; Centre D'Investigation Clinique 1402, University Hospital of Poitiers, 2 rue de la Milétrie, Poitiers F-86021, France; Cardiology Department, Hôpital la Pitié Salpétrière, 47-83 Boulevard de l'Hôpital, Paris 75013, France; Cardiology Department, University Hospital of Poitiers, 2 rue de la Milétrie, Poitiers F-86021, France; Cardiology Department, Hôpital Privé du Confluent, 2-4 Rue Éric Tabarly, Nantes 44000, France; Cardiology Department, Hôpital la Pitié Salpétrière, 47-83 Boulevard de l'Hôpital, Paris 75013, France; Cardiology Department, University Hospital Grenoble Alpes, Avenue des Maquis du Grésivaudan, Grenoble 38043, France; Cardiology Department, Hospital of Lorient, 5 Avenue Choiseul, Lorient 56322, France; Cardiology Department, University Hospital of Poitiers, 2 rue de la Milétrie, Poitiers F-86021, France; Cardiology Department, St. George's University of London, Cranmer Terrace, London SW17 OQT, UK; Cardiology Department, University Hospital of Brest, Boulevard Tanguy Prigent, Brest 29200, France; Cardiology Department, University Hospital of Nantes, boulevard Jacques-Monod Saint-Herblain, Nantes Cedex 1 44093, France; Division of Cardiology, European Georges Pompidou Hospital, 20 Rue Leblanc, Paris Cedex 15 750908, France; Université Paris Cité, PARCC, INSERM U970, Paris Cedex 15 75957, France; Cardiology Department, University Hospital of Tours, Avenue de la République, Chambray-lès-Tours 37170, France; Centre D'Investigation Clinique 1402, University Hospital of Poitiers, 2 rue de la Milétrie, Poitiers F-86021, France; Cardiology Department, University Hospital of Nantes, boulevard Jacques-Monod Saint-Herblain, Nantes Cedex 1 44093, France; Cardiology Department, University Hospital of Poitiers, 2 rue de la Milétrie, Poitiers F-86021, France

**Keywords:** Heart failure, Remote monitoring, Pre-emptive action, Mortality

## Abstract

**Aims:**

HeartLogic, an algorithm in implanted devices, predicts heart failure (HF) episodes via a remotely monitored index, aiding proactive congestion treatment to prevent acute decompensation. This study assessed the efficacy and safety of pre-emptive HF management using the HeartLogic index.

**Methods and results:**

The HeartLogic France Cohort Study is a prospective multicentre investigation involving 310 HF patients with implanted cardioverter defibrillators enrolled from 10 French centres. The HeartLogic™ index was monitored for 12 months, and when the index reached ≥16, patients were contacted to adjust HF treatment. The primary endpoint was unscheduled hospitalization for HF. An independent blinded committee adjudicated the events. A total of 309 patients (65 ± 10 years old; 83.5% male, left ventricular ejection fraction 31 ± 9%) were included in the analysis. Ischemic cardiomyopathy was present in 61.2% (*n* = 189) of the patients, and 52.8% (*n* = 163) had a cardiac resynchronization therapy device. During follow-up, 406 alerts occurred in 158 patients (1.33 alerts/patient-year), resulting in treatment modification in 39.6% of alerts. A total of 24 unplanned HF hospitalizations occurred in 19 (6.1%) patients. Sixteen (66.7%) of those hospitalizations were preceded by a HeartLogic alert, of which 14 (87.5%) triggered a drug modification before hospitalization. Eighteen deaths (5.8%) occurred, including 10 due to cardiovascular causes. An increase in creatinine ≥30% and symptomatic hypotension were reported in two patients. Hyperkalemia and hyponatremia were not observed.

**Conclusion:**

Pre-emptive HF management guided by the HeartLogic index was associated with low rates of unplanned hospitalizations and adverse events over a 12-month follow-up period.

**Study registration:**

ClinicalTrials.gov, ID: NCT04619888.

## Graphical Abstract

**Graphical Abstract:**
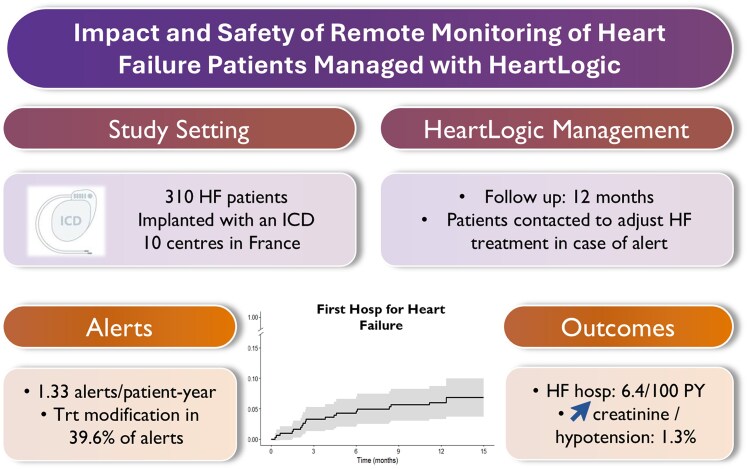

## Introduction

Heart failure (HF) is a global epidemic affecting >50 million people worldwide.^1^ Despite significant progress in drug and device therapy,^2^ it is associated with substantial morbidity and has a mortality rate of almost 50% 5 years post-diagnosis.^3^ Moreover, HF is associated with a high readmission rate, which is estimated to be >33% within the first year after diagnosis.^4^ HF places a heavy economic burden on healthcare providers, half of which is related to inpatient admission.^5^

The substantial impact of HF on both the individual patient, and its wider socioeconomic impact, together with the growth of the HF population, has prompted efforts to identify technologies to predict acute HF episodes with the aim of preventing hospitalization.^6^ The HeartLogic algorithm, combines data from several biosensors incorporated in implantable cardioverter defibrillators. It allows remote monitoring of the HF status through an index to allow the possibility of therapeutic adjustments.^7^ While the Multisense study validated the algorithm, few studies have assessed it in clinical practice to date.^8^ The Manage HF study was performed in a high-risk US population and showed relatively high alert frequency.^9^ Further investigation into the implementation of HeartLogic algorithm is necessary to understand how to integrate this remote monitoring tool in the management of patients with HF.

The aim of the HeartLogic France Cohort Study was to provide real-world data on the unplanned HF hospitalization incidence and security of pre-emptive HF management, guided by the HeartLogic index.

## Methods

### Study design

The design of the HeartLogic France Cohort Study has been described previously.^10^ The HeartLogic France Cohort Study was a prospective single-arm observational study with the main goal of assessing the annual rate of unscheduled hospitalizations for HF in a cohort of patients with HF, managed with the HeartLogic algorithm. Ten centers, including university hospitals, tertiary centers, and private clinics, participated. This study was approved by the Medical Ethics Committee ‘Centre de Protection des Personnes Sud Mediterranée IV ‘under the registration ID CRB 20202-A01835-34. The study was conducted in accordance with the Declaration of Helsinki and was registered at ClinicalTrials.gov (ID NCT04619888).

Patients ≥18 years of age were included if they met all the following criteria: (i) History of HF [left ventricular ejection fraction ≤40%, or at least one episode of clinical HF with NT-pro brain natriuretic peptide (NT-proBNP) blood concentration ≥450 ng/L] and (ii) Implantation of a cardiac defibrillator, with or without resynchronization, utilizing the HeartLogic algorithm (Resonate device family, Boston Scientific).

Patients with the following criteria were not included: (i) Concomitant HF device other than cardiac resynchronization, such as a ventricular assist device or cardiac contractility modulation device; (ii) Planned heart transplant, or patients with a heart transplant; (iii) Glomerular filtration rate <30 mL/min/m^2^ or receiving dialysis; (iv) Life expectancy ≤ 6 months; (v) Remote monitoring of HeartLogic not possible; (vi) Non-compliance with HF medications; and (vii) Patients with a mechanical heart valve.

### Study protocol

Patients were enrolled within the month following cardioverter defibrillator implantation and were followed up every 3 months for 1 year. Data on unscheduled hospitalization for HF, atrial and ventricular arrhythmia, and medication history were collected at each visit.

For each patient, the HeartLogic index was monitored remotely on the Latitude platform on a weekly basis for 12 months. If the index value was ≥16, the investigator assessed the clinical status of the patient. If there was no obvious reason for a false alert, diuretics were increased by 50–100% for 3 to 5 days.^10^ Re-evaluation of the clinical status of the HeartLogic index and renal function was recommended after 3–5 days of diuretic increase. When an alert was received, details of the treatment adjustment were collected or where treatment remained unaltered, the reasons were recorded. An alert was considered to precede an event if the patient was in an alert state at any point within the 34-day window prior to the index event, irrespective of the alert onset date.

### Study endpoints

The primary endpoint was the occurrence of an unscheduled hospitalization for ≥1 day due to HF decompensation, defined by the presence of HF signs or symptoms with evidence of HF on diagnostic evaluation on at least one test (elevated natriuretic peptides, radiological or echocardiographic evidence) requiring an increase in diuretic treatment.^11^ Scheduled hospitalization for routine follow-up, pre-transplant assessment or iron injection were not considered unscheduled hospitalization.

The secondary endpoints were all-cause death, death from cardiovascular causes, death from HF, unplanned hospitalizations for ventricular and atrial arrhythmia, number of days of hospitalization for HF, ventricular or atrial arrhythmia, and a safety endpoint (combining a creatinine level increase ≥30%, a potassium level change ≥30%, a sodium level decrease ≥30% between the HeartLogic alert and the end of the diuretic treatment, or the occurrence of symptomatic hypotension related to diuretic treatment initiation or increase). Unexplained alerts were defined as alerts that were not followed by treatment modification or by an hospitalization.

Outcomes were adjudicated in a blinded fashion according to the pre-specified definitions by two independent cardiologists. Data management and statistical analyses were performed at Centre d'Investigation Clinique 1402 INSERM at the University Hospital of Poitiers, Poitiers, France.

### Statistical considerations

Assuming an incidence of rehospitalization for HF of 10% for one year of follow-up, we planned to include 310 patients to obtain an absolute precision of 3.5%, with an alpha risk of 5% and a 10% loss of follow-up.

Quantitative variables were presented as mean ± standard deviation or median and interquartile range (IQR) in the case of non-normal distribution. Qualitative variables were presented as number and percentage. The baseline characteristics of patients with or without HeartLogic alerts were compared using the χ^2^ test or Student’s *t*-test.

The one-year probabilities of first rehospitalization for HF, first rehospitalization for atrial arrhythmia, first rehospitalization for ventricular arrhythmia, all-cause death, death from cardiovascular cause and death from HF were expressed as cumulative incidence and 95% confidence interval (CI) at 6 and 12 months, and as incidence density with 95% CI expressed per 100 person-years. Cumulative incidences of first rehospitalization and all-cause, cardiovascular, and HF-related deaths were described using Kaplan–Meier curves. Alert rates were expressed as alerts per patient-year and were calculated as the ratio between the total number of alerts and the cumulative follow-up duration.

All patients included in the study were considered in the data analysis, provided they had not withdrawn their consent or unless HeartLogic data transmissions were not possible. No imputation was performed for the missing data. No interim analysis was planned, and no correction for multiplicity was done. Statistical analyses were performed using R (version 4.0.4). A two-sided *P*-value < 0.05 was considered statistically significant.

## Results

### Baseline characteristics

**Figure 1:**
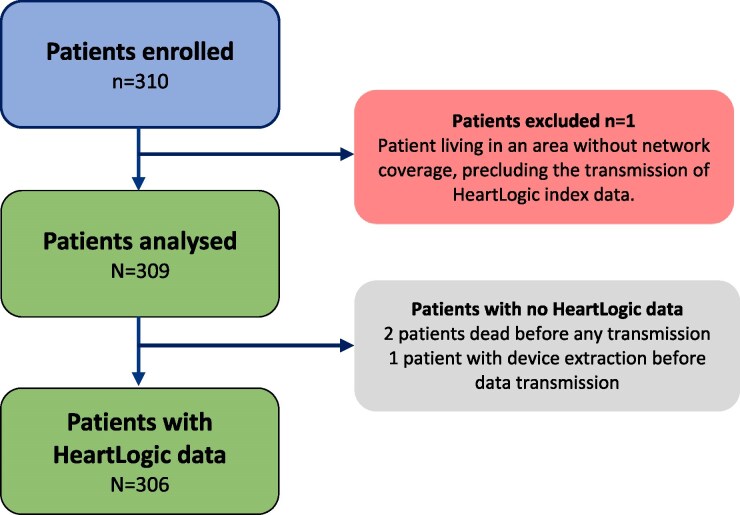
Flow chart. Among the 310 patients included, one patient was excluded from the analysis because living in an area without coverage, preventing transmission of the HeartLogic index. Three other patients were included in the analysis but had no HeartLogic data available: two patients died, and one patient underwent device extraction before the first transmission.

A total of 310 patients were enrolled between March 2021 and July 2023 (*Figure 1*). One patient, who met an exclusion criterion by residing in an area without telephone coverage, was excluded from the analysis, as this prevented any HeartLogic data transmission.

Among the 309 remaining patients, the mean age of the participants was 65 ± 10 years, and 258 (83.5%) were male. Hypertension, ischemic heart disease, atrial fibrillation, and chronic renal failure were present in 175 (56.6%), 189 (61.2%), 86 (27.8%), and 27 (8.7%) patients, respectively (*Table 1*). On electrocardiogram, the mean heart rate and QRS width were 71 ± 18 beats/min and 136 ± 34 ms, respectively. The mean left ventricular ejection fraction was 31 ± 9%, and the median NT-proBNP level was 1000 (IQR 394–2299) ng/L.

**Table 1:** Baseline population characteristics

	All patients (*n* = 309)
Male sex, *n* (%)	258 (83.5%)
Age (year)	65 ± 10
Body mass index (kg/m^2^)	27.1 ± 5.0
New York Heart Association class, *n* (%)	
I/II	231 (74.8%)
III/IV	78 (25.2%)
*Medical history*	
Hypertension, *n* (%)	175 (56.6%)
Diabetes, *n* (%)	90 (29.1%)
Hypercholesterolemia, *n* (%)	160 (51.8%)
Active smoking, *n* (%)	48 (15.5%)
Ischemic heart disease, *n* (%)	189 (61.2%)
Valvular heart disease, *n* (%)	29 (9.4%)
Dilated cardiomyopathy, *n* (%)	97 (31.4%)
Atrial fibrillation, *n* (%)	86 (27.8%)
Sleep apnea syndrome, *n* (%)	34 (11.0%)
Chronic renal failure, *n* (%)	27 (8.7%)
Nt pro BNP (ng/L)	1000 (394–2299)
Creatinine (µmol/L)	107 ± 46
Haemoglobin (g/dL)	13.8 ± 1.7
Left ventricular ejection fraction (%)	31 ± 9
Heart rate (bpm)	71 ± 18
QRS width (ms)	136 ± 34
Bundle branch block, *n* (%)	
Right	38 (12.4%)
Left	145 (47.2%)
CRT device, *n* (%)	163 (52.8%)
*Baseline drugs*	
Beta-blockers, *n* (%)	274 (88.7%)
ACE-inhibitor, ARB, *n* (%)	100 (32.4%)
ARNi, *n* (%)	177 (57.3%)
SGLT2 inhibitor, *n* (%)	182 (58.9%)
MRA, *n* (%)	180 (58.3%)
Diuretics, *n* (%)	176 (57.0%)
Anticoagulants, *n* (%)	143 (46.3%)
Amiodarone, *n* (%)	84 (27.2%)

ACE, angiotensin converting enzyme; ARB, angiotensin II receptor blockers; ARNi, angiotensin receptor-neprilysin inhibitor, CRT, cardiac resynchronization therapy, MRA, mineralocorticoid receptor antagonists, SGLT2, sodium-glucose co-transporter 2.

Regarding medication therapy, 274 (88.7%) patients were on beta-blockers, 277 (89.7%) on angiotensin converting enzyme-inhibitors, angiotensin II receptor blockers, or sacubitril/valsartan, 182 (58.9%) on sodium-glucose co-transporter 2 inhibitors, and 180 (58.3%) on mineralocorticoid receptor antagonists at baseline.

Fifty (16.2%) patients were implanted with a single-chamber implantable cardioverter defibrillator, 96 (31.1%) with a dual-chamber implantable cardioverter defibrillator, and 163 (52.7%) with a cardiac resynchronization therapy device.

### HeartLogic alert management

Among the 309 patients analysed, 306 had HeartLogic transmissions. One patient underwent implantable cardioverter defibrillator extraction before the first transmission, and two patients died before the first HeartLogic transmission (*Figure 1*). The first HeartLogic index calculation was available 39 ± 8 days after device implantation and for a median period of 47 (IQR 46–50) weeks.

Overall, 406 alerts (1.33 alerts/patient-year in the overall population and 2.55 alerts/patient-year in the group of patients with at least one alert) were generated in 158 patients. The median duration of alerts was 18 days (IQR 8–30 days). The cumulative time spent in the alert state, relative to the total duration of HeartLogic monitoring, was 0.9% (IQR 0.0–16.0%) in the overall cohort and 15.6% (IQR 6.6–25.8%) among patients who experienced at least one alert. Globally, the HeartLogic index peaked at the 11th week and then declined and remained stable over time (see Supplementary material online, *Figure S1*). Patients with at least one HeartLogic alert were more likely to have hypertension (63.9% vs. 49.0%; *P* = 0.008), atrial fibrillation (39.2% vs. 15.9%; *P* = 0.0001), sleep apnea (15.2% vs. 6.6%; *P* = 0.016) or right bundle branch block (18.4% vs. 6.0%; *P* = 0.004), and had higher heart rate (73 ± 19 beats/min vs. 69 ± 16 beats/min; *P* = 0.028) and NT-proBNP levels [1445 (IQR 590–3310) ng/L vs. 758 (IQR 312-1738) ng/L; *P* = 0.0001] than those without any alert (see Supplementary material online, *Table S1*).

**Figure 2:**
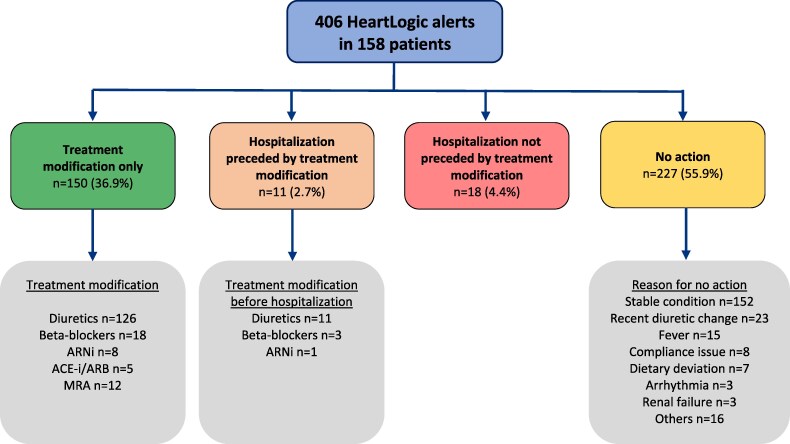
HeartLogic alerts management. Among the 309 patients, 158 experienced a total of 406 alerts. These alerts resulted in no action for 55.9% of cases, medication adjustment alone in 36.9%, and hospitalization in 7.1%. *n* = number of alert cases. ACE, Angiotensin converting enzyme; ARB, Angiotensin II receptor blockers; ARNI, Angiotensin receptor-neprilysin inhibitor; MRA, Mineralocorticoid receptor antagonists.

Of the 406 alerts, 150 (36.9%) prompted an isolated treatment adjustment, and 29 (7.1%) led to hospitalization, independent of any preceding treatment changes (*Figure 2*). The main reasons for the absence of treatment modification (227 cases) were that the patient was considered stable (152 cases, 66.9%), recent diuretic treatment modification (23 cases, 10.1%), compliance issues (8 cases, 3.5%), and deviation from a low-sodium diet (7 cases, 3.1%). Overall, the incidence of unexplained alerts was 0.74 alert per patient-year.

### Outcomes

**Figure 3:**
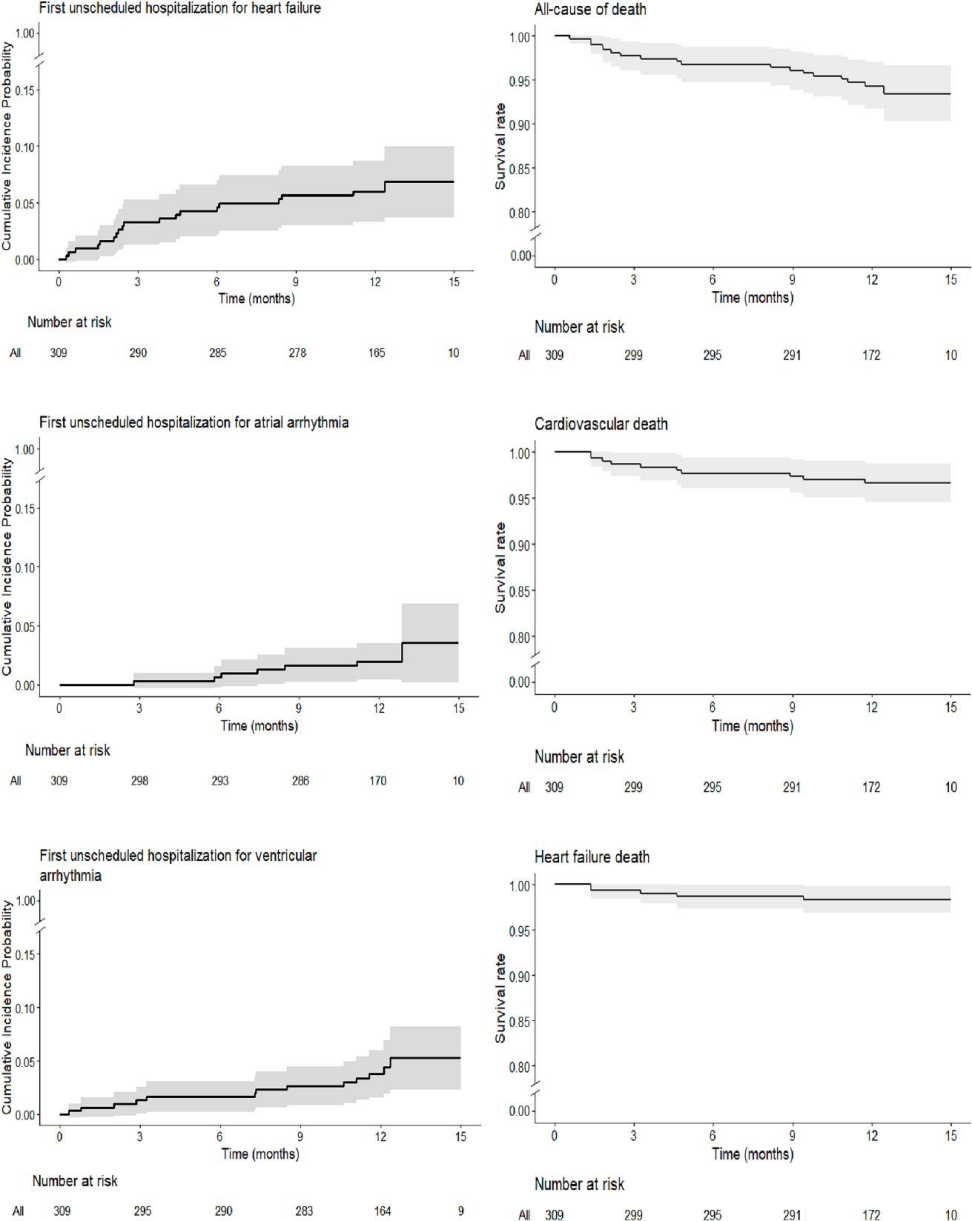
Kaplan Meier curves for unscheduled hospitalizations for heart failure, all-cause death, cardiovascular death, heart failure death, unscheduled hospitalization for ventricular arrhythmia and atrial arrhythmia in the overall population. The cumulative incidences of first unplanned hospitalizations at 6 and 12 months were 4.2% [95% CI: 2.0–6.5] and 5.9% [95% CI: 3.3–8.6], respectively.

**Figure 4:**
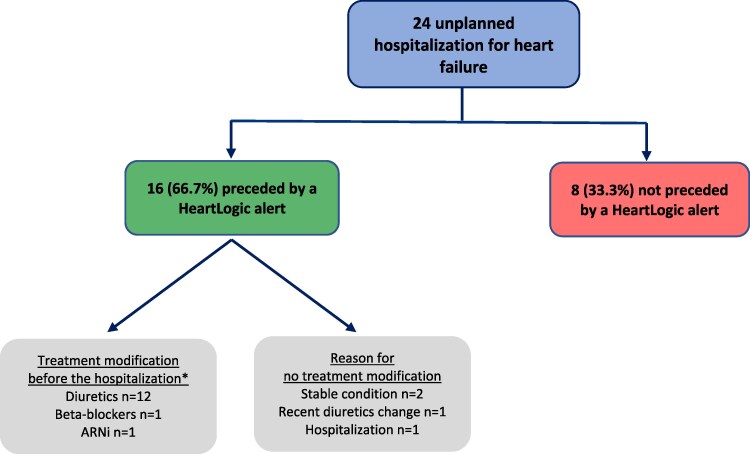
Analysis of unplanned hospitalizations for heart failure. Among the 24 unplanned hospitalizations for heart failure, 33.3% were not preceded by a HeartLogic alert. *One patient underwent a dosage adjustment of both diuretics and beta-blockers, while another patient had modifications to both diuretics and the sacubitril/valsartan therapy. ARNi, Angiotensin receptor-neprilysin inhibitor.

During a median follow-up of 12.2 months [95% CI: 12.1–12.3], a total of 24 unplanned HF hospitalizations occurred in 19 patients (*Table 2*), providing an incidence density of 6.41 [95% CI: 3.68–9.64] per 100 person-years. The cumulative incidences of first unplanned hospitalization at 6 and 12 months were 4.2% [95% CI: 2.0–6.5] and 5.9% [95% CI: 3.3–8.6], respectively (*Figure 3*). The total number of days of hospitalization for HF was 261, with a median duration of 9 (IQR: 4–14) days. Among the 24 unplanned HF hospitalizations, 16 (66.7%) were preceded by a HeartLogic alert, of which 14 (87.5%) triggered drug modifications before hospitalization (*Figure 4*).

**Table 2:** Description of hospitalizations for heart failure, ventricular arrhythmia and atria arrhythmia

	Number of patients with at least one hospitalizations	Total number of event	Number of event with prior alert	Number of event without prior alert	Days of hospitalization, *Median (IQR)*
Unscheduled hospitalization for HF	19	24^a^	16	8	9 (4–14)
Unscheduled hospitalization for ventricular arrhythmia	13	16^b^	6	10	6 (3–15)
Unscheduled hospitalization for atrial arrhythmia	7	7^c^	3	4	5 (2–7)
All-cause death	/	18	6	12	/

HF, heart failure.

^a^14 patients had one unscheduled hospitalization for HF and 5 patients had two unscheduled hospitalization for HF.

^b^10 patients had one unscheduled hospitalization for ventricular arrhythmia and 3 patients had two unscheduled hospitalization for ventricular arrhythmia.

^c^7 patients had one unscheduled hospitalization for atrial arrhythmia.

Unscheduled hospitalization for ventricular arrhythmia occurred 16 times in 13 patients [incidence, 4.32 (95% CI: 2.29–6.77) per 100 person-years] for a total of 216 days. Unscheduled hospitalization for atrial arrhythmia occurred seven times in seven patients [incidence 2.29 (95% CI: 0.96–4.30) per 100 person-years] for a total of 44 days of hospitalization (*Table 2*).

A total of 18 patients died, leading to an all-cause mortality incidence density of 5.91 [95% CI: 3.48–9.22] per 100 person-years. The cumulative incidence of all-cause death at 6 and 12 months was 3.3% [95% CI: 1.3–5.2] and 5.7% [95% CI: 3.0–8.3], respectively. Among them, 10 were due to cardiovascular causes [incidence density 3.26 (95% CI: 1.60–5.56) per 100 person-years] and 5 due to HF [incidence density 1.63 (95% CI 0.32–3.27) per 100 person-years]. The survival curves are shown in *Figure 3*.

The incidence density of all events (including unplanned hospitalizations for HF, hospitalizations for atrial or ventricular arrhythmia, and all-cause death) was 47.64 events per 100 person-years during the *in-alert* state, compared with 6.65 events per 100 person-years during the *out-of-alert* state, yielding an event rate ratio of 7.2.

At baseline, an NT-proBNP level above 1000 pg/mL was associated with an incidence density of 25.21 events per 100 person-years, compared with 10.96 events per 100 person-years below this threshold, corresponding to an event rate ratio of 2.3.

The overall rate of safety endpoint was 1.29%. An increase in creatinine ≥30% and symptomatic hypotension were both reported in two patients. Dyskalemia and hyponatremia were not observed.

## Discussion

The HeartLogic France Cohort Study assessed the implementation of the multiparametric HeartLogic algorithm for remote HF monitoring in routine clinical practice. The primary findings revealed an unexpectedly low incidence of unplanned HF hospitalization during the follow-up period. Notably, fewer than 50% of alerts resulted in treatment modifications, predominantly because the patients were clinically assessed as stable or improving. Importantly, HeartLogic alert-triggered interventions, such as diuretic up titration, were well tolerated without significant safety concerns.

### Alerts

The observed alert rate was 1.33 alerts/patient-year, lower than reported in the Manage-HF study (1.76 alerts/patient-year) but higher compared to other European cohorts (Calò *et al*. 0.76 alert per patient-year,^12^ Santini *et al*. 0.93 alerts per patient-year,^13^ Llewellyn *et al*. 0.93 alerts per patient-year,^14^ Feijen *et al*. 0.88 alerts per patient-year^15^). In the present study, two-thirds of HF hospitalizations were preceded by a HeartLogic alert (sensitivity = 67%), consistent with findings from the MultiSENSE trial, which demonstrated an approximately 70% sensitivity for predicting HF decompensation. However, the proportion of ‘unheralded’ HF events was lower in other studies (17% in Manage HF,^9^ 21% in Calò *et al*.^12^ 0% in Llewellyn *et al.*^14^) Variability in alert frequency and sensitivity across studies likely reflects differences in patient characteristics, HF severity, baseline medication use, and the inclusion criteria. Indeed, hospitalizations for HF within one year was not a mandatory inclusion criterion in our study.

### Outcomes

Regarding clinical outcomes, the rate of unplanned HF hospitalizations (0.0641 per patient-year) was notably lower compared to the Manage-HF study^9^ (0.26 per patient-year) and other European reports (Santini *et al*.: 0.15;^13^ Calò *et al*.: 0.10;^12^ Llewellyn *et al*.: 0.07).^14^ Conversely, mortality rates were comparable across studies (0.0591 per patient-year in our study vs. 0.05–0.08 in others).^9,12,14^ Several factors might explain these differences. First, our population was managed with contemporary, optimized pharmacotherapy for HF.^16^ Notably, the utilization of medications such as mineralocorticoid receptor antagonists and sodium-glucose co-transporter 2 inhibitors was high at baseline (∼60%) compared to other studies.^17,18^ These therapies independently lower HF hospitalizations risk.^19–22^ Second, the proactive approach adopted in the study, with prompt clinical intervention upon receiving alerts, might have enhanced adherence, improved patient self-management, and consequently reduced hospitalizations. Third, the inclusion criteria and patient profile likely contributed to the lower event rates compared to studies like the MultiSENSE trial, which enrolled a higher-risk cohort with recent HF admissions.

### Implementation of HeartLogic in real-life

The implementation of an algorithm intended to avoid HF-related hospitalizations based on the pre-emptive increase of diuretics is not yet advocated in current international guidelines; however, if its usefulness is confirmed, it may change the current paradigm in HF management. Several factors must be considered when implementing HeartLogic in routine clinical practice. Adequate staffing of co-ordinated remote monitoring and HF management teams is crucial, given that even low-alert rates can generate substantial workloads. A recent publication estimated that 257 h were required for the HF management of 212 patients during 1 year.^14^ Second, the establishment of clear and standardized clinical protocols for responding to alerts remains essential to minimize practice variability. Despite the availability of such protocols, our study, as well as prior investigations, including Manage-HF, demonstrated substantial heterogeneity in clinical responses.^9^ Evidence supporting the safety and effectiveness of early therapeutic interventions in asymptomatic or minimally symptomatic patients will facilitate clinician acceptance and standardized management. Finally, patient compliance and education are indispensable to ensuring alert efficacy and preventing unnecessary hospitalizations.^16^ Looking ahead, continued advances in implantable cardiac monitors and wearable biosensors hold promise for expanding remote HF management by enabling continuous, multiparametric monitoring and early intervention beyond traditional clinical settings.^23^ For example, a minimally invasive insertable cardiac monitor has already demonstrated the ability to detect impending HF decompensation even in patients who are ineligible for conventional implantable devices, suggesting that such technologies could broaden the accessibility of proactive HF care.^24^ Nevertheless, optimizing patient selection for this device remains an important consideration. Prior investigations have demonstrated that patients who do not trigger HeartLogic alerts typically exhibit more favourable cardiac profiles, including higher left ventricular ejection fraction and a lower burden of HF symptoms, compared with those who trigger alerts more frequently.^25^ Consistently, our findings suggest that patients with less advanced HF, in the absence of hypertension and atrial fibrillation, were less likely to generate alerts.

### Strengths and limitations

The strengths of our study include its prospective multicenter design, independent adjudication of clinical events, and uniform implementation of the HeartLogic management algorithm. Nevertheless, the absence of a control group represents a significant limitation, preventing the definitive attribution of clinical outcomes solely to HeartLogic monitoring. Additionally, the study cohort did not include patients with physiological pacing systems, limiting the generalizability of the results. Finally, although their occurrence was expected to be infrequent, unscheduled outpatient visits necessitating initiation or intensification of HF therapy were not captured in the study.

## Conclusion

In conclusion, the HeartLogic France Cohort Study demonstrated the feasibility and safety of implementing HeartLogic-guided HF management, as reflected by notably low rates of hospitalizations. Future randomized controlled trials are necessary to definitively establish the algorithm’s effectiveness in reducing HF-related hospitalizations and its broader applicability in routine clinical practice.

## Supplementary Material

ztaf133_Supplementary_Data

## Data Availability

The data underlying this article will be shared on reasonable request to the corresponding author.
