# Supplementary material for: Impact and safety of remote monitoring of heart failure patients managed with the HeartLogic algorithm: the HeartLogic France Cohort Study
Source: Eur Heart J Digit Health. 2025 Nov 13;7(2):ztaf133. doi: 10.1093/ehjdh/ztaf133 (PMC12853121; doi:10.1093/ehjdh/ztaf133)
Supplement: ztaf133_Supplementary_Data [file ztaf133_supplementary_data.docx]

**SUPPLEMENTARY FIGURE 1: Description of HeartLogic index evolution over time**

**
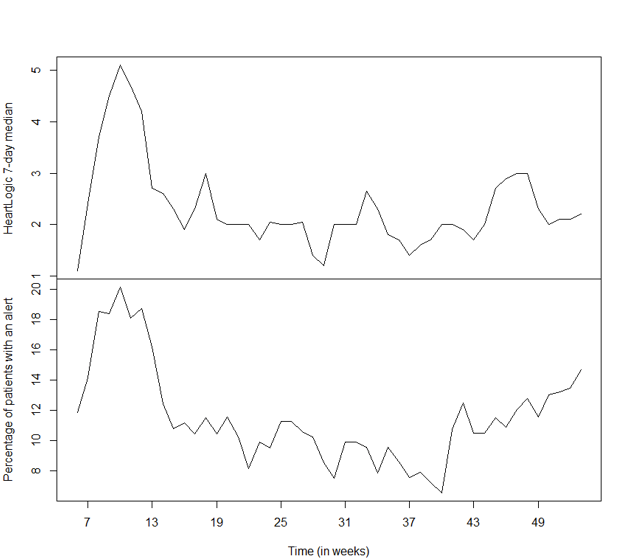
**

**SUPPEMENTARY TABLE 1 : Baseline population characteristics and outcomes according to the presence of alert**

|  | **Patients with no alert**  **(n=151)** | **Patients with at least one alert (n=158)** | **P value** |
| --- | --- | --- | --- |
| Men sex, n (%) | 124 (82.1%) | 134 (84.8%) | 0. 524 |
| Age (year) | 64 ± 10 | 66 ± 10 | 0.148 |
| Body mass index (kg/m2) | 26.8 ± 4.8 | 27.4 ± 5.2 | 0.322 |
| New York Heart Association class, n (%) |  |  | 0.180 |
| -I/II | 118 (78.1%) | 113 (71.5%) |  |
| -III/IV | 33 (21.9%) | 45 (28.5%) |  |
|  |  |  |  |
| *Medical history* |  |  |  |
| Hypertension, n (%) | 74 (49.0%) | 101 (63.9%) | 0.008 |
| Diabetes, n (%) | 42 (27.8%) | 48 (30.4%) | 0.620 |
| Hypercholesterolemia, n (%) | 74 (49.0%) | 86 (54.4%) | 0.340 |
| Active smoking, n (%) | 19 (12.6%) | 29 (18.4%) | 0.161 |
| Ischemic heart disease, n (%) | 88 (58.3%) | 101 (63.9%) | 0.309 |
| Valvular heart disease, n (%) | 12 (7.9%) | 17 (10.8%) | 0.397 |
| Dilated cardiomyopathy, n (%) | 53 (35.1%) | 44 (27.8%) | 0.170 |
| Atrial fibrillation, n (%) | 24 (15.9%) | 62 (39.2%) | <0.0001 |
| Sleep apnea syndrome, n (%) | 10 (6.6%) | 24 (15.2%) | 0.016 |
| Chronic renal failure, n (%) | 15 (9.9%) | 12 (7.6%) | 0.467 |
|  |  |  |  |
| Nt pro BNP (ng/L) | 758 (312-1738) | 1445 (590-3310) | <0.0001 |
| Creatinine (µmol/L) | 105 ± 39 | 108 ± 52 | 0.585 |
| Haemoglobin (g/dL) | 13.8 ± 1.6 | 13.7 ± 1.9 | 0.449 |
| Left ventricular ejection fraction (%) | 31 ± 9 | 31 ± 9 | 0.418 |
| Heart rate (bpm) | 69 ± 16 | 73 ± 19 | 0.028 |
| QRS width (ms) | 138 ± 34 | 134 ± 34 | 0.329 |
| Bundle branch block, n (%) |  |  | 0.004 |
| -Right | 9 (6.0%) | 29 (18.4%) |  |
| -Left | 77 (51.7%) | 68 (43.0%) |  |
|  |  |  |  |
| CRT, n (%) | 85 (56.3%) | 78 (49.4%) | 0.223 |
|  |  |  |  |
| *Baseline drugs* |  |  |  |
| Beta-blockers, n (%) | 135 (89.4%) | 139 (88.0%) | 0.692 |
| ACE-inhibitor, ARB, n (%) | 50 (33.1%) | 50 (31.6%) | 0.783 |
| ARNi, n (%) | 88 (58.3%) | 89 (56.3%) | 0.729 |
| SGLT2 inhibitor, n (%) | 96 (63.6%) | 86 (54.4%) | 0.102 |
| MRA, n (%) | 91 (60.3%) | 89 (56.3%) | 0.483 |
| Diuretics, n (%) | 81 (53.6%) | 95 (60.1%) | 0.250 |
| Anticoagulants, n (%) | 59 (39.1%) | 84 (53.2%) | 0.013 |
| Amiodarone, n (%) | 39 (25.8%) | 45 (28.5%) | 0.600 |
|  |  |  |  |
| *Outcomes* |  |  |  |
| All-cause death, *per 100 person-years* | 5.27 [1.99 ; 9.71] | 6.12 [95% CI: 2.64 ; 10.53] | 0.729 |
| Death from cardiovascular causes, *per 100 person-years* | 3.34 [0.68 ; 6.81] | 3.08 [0.62 ; 6.32] | 0.894 |
| Death from HF, *per 100 person-years* | 1.36 [0.00 ; 3.55] | 1.86 [0.00 ; 4.75] | 0.679 |
| Unplanned HF hospitalization, *per 100 person-years* | 2.02 [0.00 ; 4.95] | 10.73 [5.95 ; 16.42] | <0.0001 |
| Unscheduled hospitalization for ventricular arrhythmia, *per 100 person-years* | 2.00 [0.00 ; 4.51] | 6.27 [2.70 ; 11.00] | 0.031 |
| Unscheduled hospitalization for atrial arrhythmia, *per 100 person-years* | 1.35 [0.00 ; 3.58] | 3.13 [0.65 ; 6.20] | 0.083 |
|  | | | |

*Abbreviations: ACE = angiotensinconverting enzyme; ARB = angiotensin II receptor blockers; ARNI = angiotensin receptor-neprilysin inhibitor, CRT = cardiac resynchronization therapy, MRA = mineralocorticoid receptor antagonists, SGLT2 = sodium-glucose co-transporter 2.*
